# Transcatheter dual-device closure for symptomatic iatrogenic atrial septal defect following bioprosthetic mitral valve replacement: a case report

**DOI:** 10.3389/fmedt.2026.1847831

**Published:** 2026-06-30

**Authors:** Ping-an Lian, Shan-fu Liang, Fei Xie, Shuai Cheng, Zhan-zhan Zhu, Yun-fei Zhao, Lei Yin, Shen-wei Zhang

**Affiliations:** 1 Department of Cardiology, Seventh People’s Hospital of Zhengzhou, Zhengzhou, Henan, China; 2 Institute of Biological Therapy, Henan Academy of Innovations in Medical Science, Zhengzhou, Henan, China

**Keywords:** dual-device strategy, heart failure, iatrogenic atrial septal defect, transcatheter intervention, valve surgery

## Abstract

Iatrogenic atrial septal defect (iASD) is a relatively common complication after cardiac surgical or catheter-based procedures involving transseptal access. While most defects close spontaneously, a subset may persist and become hemodynamically significant, leading to clinical deterioration and necessitating intervention. We report a case of a large, persistent iASD that led to progressive right-sided heart failure following bioprosthetic mitral valve replacement combined with tricuspid annuloplasty. A 69-year-old male underwent bioprosthetic mitral valve replacement and tricuspid annuloplasty. Postoperatively, the patient developed a large, persistent iASD, which contributed to severe right-sided heart failure. The patient also had end-stage liver disease, portal hypertension, and severe coagulopathy, which made reoperation extremely high-risk. After heart team evaluation, a transcatheter closure approach was chosen. Initially, a 24-mm occluder was deployed, but significant residual shunting remained. A planned two-device strategy was then performed, resulting in complete closure of the defect. Post-procedure imaging confirmed the absence of residual shunt, and tricuspid regurgitation significantly improved. The patient's symptoms improved to New York Heart Association NYHA I–II, and at six-month follow-up, the device remained stable, and cardiac function had recovered. This case illustrates that in patients with large, complex, or poorly rimmed iASDs, particularly those who are not suitable candidates for reoperation, the dual-device strategy represents a safe and effective percutaneous alternative.

## Introduction

Iatrogenic atrial septal defect (iASD) most commonly occurs after transseptal puncture procedures for catheter-based structural heart interventions or intentional atrial septotomy performed during cardiac surgery, such as mitral valve operations, to access the left atrium (1, 2). The reported incidence of iASD following transcatheter procedures ranges from 24% to 50% (2, 3), whereas its occurrence after surgical valve interventions is considerably less common (1). Although most small defects tend to close spontaneously within several months, a subset of patients may develop persistent left-to-right or right-to-left shunting due to large residual defects, potentially leading to right-sided heart failure, hypoxemia, or paradoxical embolism (4). Currently, there are no evidence-based guidelines regarding iASD management. Surgical repair remains the conventional treatment for symptomatic patients; however, transcatheter closure has emerged as a safe and effective minimally invasive alternative in anatomically suitable cases, particularly among those considered high risk for redo surgery (5). In patients with advanced liver cirrhosis, concomitant coagulopathy, thrombocytopenia, and portal hypertension substantially increase perioperative bleeding and postoperative complications. Additionally, the hyperdynamic circulatory state associated with cirrhosis may further exacerbate the hemodynamic consequences of iASD. Here, we report a complex case of a large symptomatic iASD that developed following bioprosthetic mitral valve replacement in a patient with decompensated cirrhosis and severe pancytopenia, successfully treated with a sequential dual-device transcatheter closure strategy. This report aims to provide a practical, step-by-step operational approach for other operators to refer to when a single device fails to achieve complete closure of a similarly complex iatrogenic atrial septal defect.

## Case description

A 69-year-old man presented with progressively worsening exertional dyspnea corresponding to NYHA functional class III. His medical history was notable for hepatitis B–related cirrhosis complicated by esophageal varices, portal hypertension, splenomegaly, and hypersplenism resulting in leukopenia and thrombocytopenia (WBC 1.97 × 10⁹/L, hemoglobin 96 g/L, and platelet count 74 × 10⁹/L). Liver transaminases and bilirubin levels were within normal ranges, whereas coagulation testing revealed prolonged prothrombin time (PT 15.5 s). Preoperative transthoracic echocardiography (TTE) demonstrated severe mitral and tricuspid regurgitation, pulmonary hypertension (estimated systolic pulmonary artery pressure 55 mmHg), and marked biatrial enlargement, while left ventricular systolic function was preserved. Electrocardiography revealed atrial fibrillation. After multidisciplinary heart team evaluation, the patient underwent elective cardiac surgery in May 2024, consisting of bioprosthetic mitral valve replacement (27-mm Puraphy valve) and tricuspid annuloplasty (30-mm King's ring) via a median sternotomy and transseptal approach, along with left atrial appendage excision. The intraoperative and early postoperative courses were uneventful. He was discharged on rivaroxaban 15 mg once daily for anticoagulation, in addition to guideline-directed heart failure therapy.

**Figure 1:**
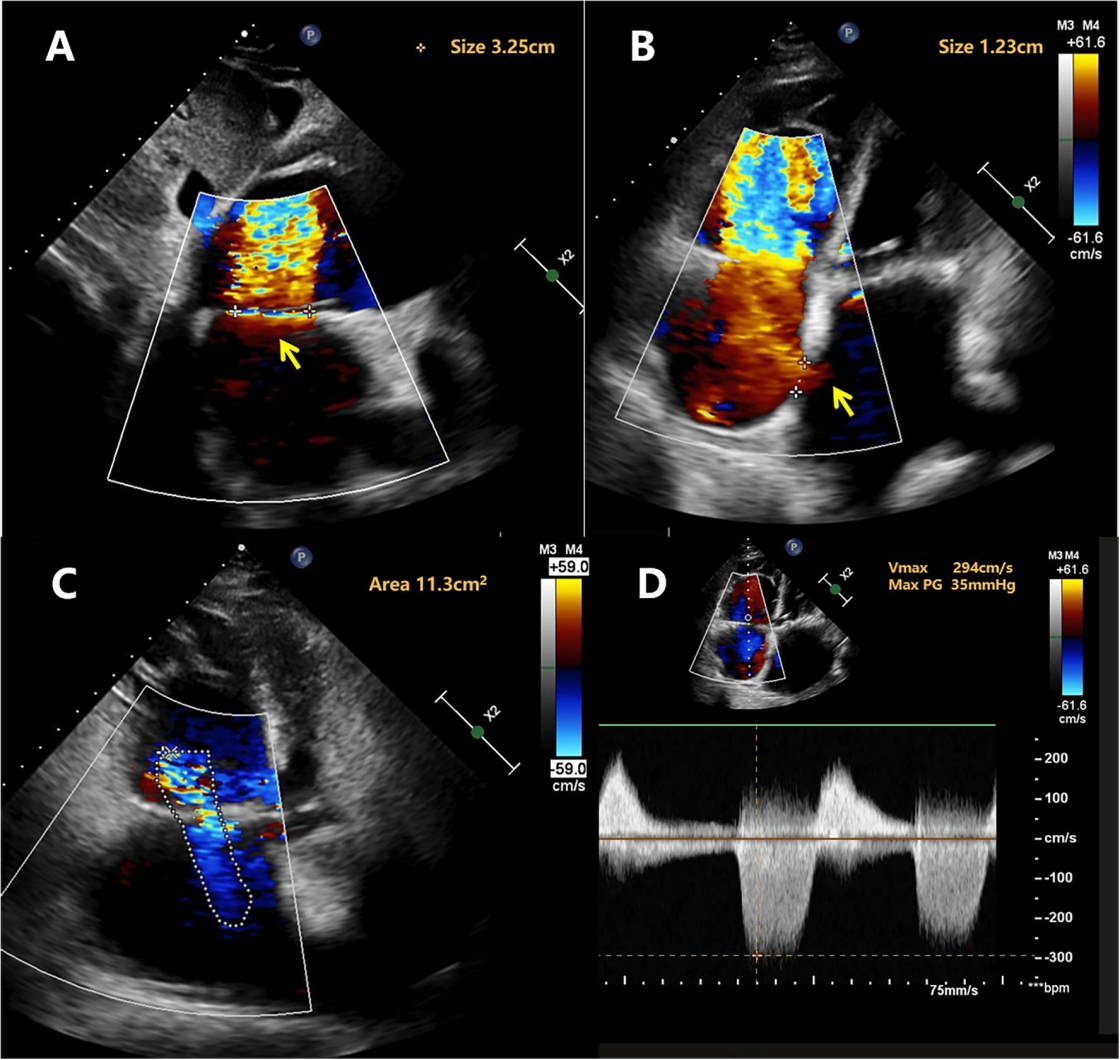
Echocardiographic findings demonstrating the atrial septal defect with significant left-to-right shunting. **(A)** Extensive left-to-right flow across the mid-atrial septum, with a defect length of approximately 3.25 cm; **(B)** Maximal shunt orifice measuring approximately 1.23 cm; **(C)** Tracing of the atrial septal defect region showing an effective shunt area of approximately 11.3 cm^2^; **(D)** Pulsed-wave Doppler across the tricuspid level showing a peak shunt velocity of approximately 294 cm/s, corresponding to a pressure gradient of about 35 mmHg.

Four months after mitral valve replacement, the patient was rehospitalized due to worsening exertional dyspnea and recurrent lower-extremity edema. Physical examination revealed an irregularly irregular pulse, jugular venous distension, and bilateral pitting edema below the knees. Repeat transthoracic echocardiography (TTE) demonstrated normal prosthetic mitral valve function with a mean transmitral gradient of 7 mmHg; however, multiple left-to-right shunt jets were identified across the mid-atrial septum, with an overall defect length of approximately 33 mm and a maximal shunt orifice of about 12 mm. The right atrium and right ventricle were markedly dilated, accompanied by severe tricuspid regurgitation (Figure 1).

Although the atrial septotomy was routinely closed with interrupted sutures at the end of the index surgery, and postoperative transthoracic echocardiography showed no evidence of shunting, the subsequent increase in right heart volume load and liver disease-related coagulopathy may have subjected the atrial septal suture line to uneven tension. This likely led to gradual suture loosening or partial disruption, resulting in the delayed appearance of a large, hemodynamically significant iatrogenic atrial septal defect. The clinical deterioration primarily attributed to substantial left-to-right shunting, resulting in increased right-sided volume overload and worsening of the patient's pre-existing right ventricular dysfunction, ultimately leading to right-sided heart failure. The patient exhibited poor response to conventional diuretic therapy and standard heart failure management. Given the presence of advanced cirrhosis, portal hypertension, and severe thrombocytopenia, the perioperative risk associated with repeat open surgical repair—particularly the risk of major bleeding—was deemed exceedingly high. After multidisciplinary discussion involving cardiology, cardiac surgery, and hepatology, transcatheter closure of the iASD was selected as the preferred therapeutic approach. Based on pre-procedural echocardiographic assessment showing a maximal shunt orifice of approximately 1.23 cm, a stepwise closure strategy was planned: implantation of a 24-mm occluder as the initial device, with completion of the procedure if intraoperative echocardiography confirmed no more than trivial or mild residual shunt; however, if a moderate-to-large residual shunt was observed, a dual-device strategy would be initiated, involving partial retrieval of the initial occluder and deployment of a second device through an independent access pathway. This staged approach was designed to avoid potential complications associated with oversizing a single device, such as aortic root impingement, atrioventricular conduction disturbance, or interference with the bioprosthetic mitral valve.

**Figure 2:**
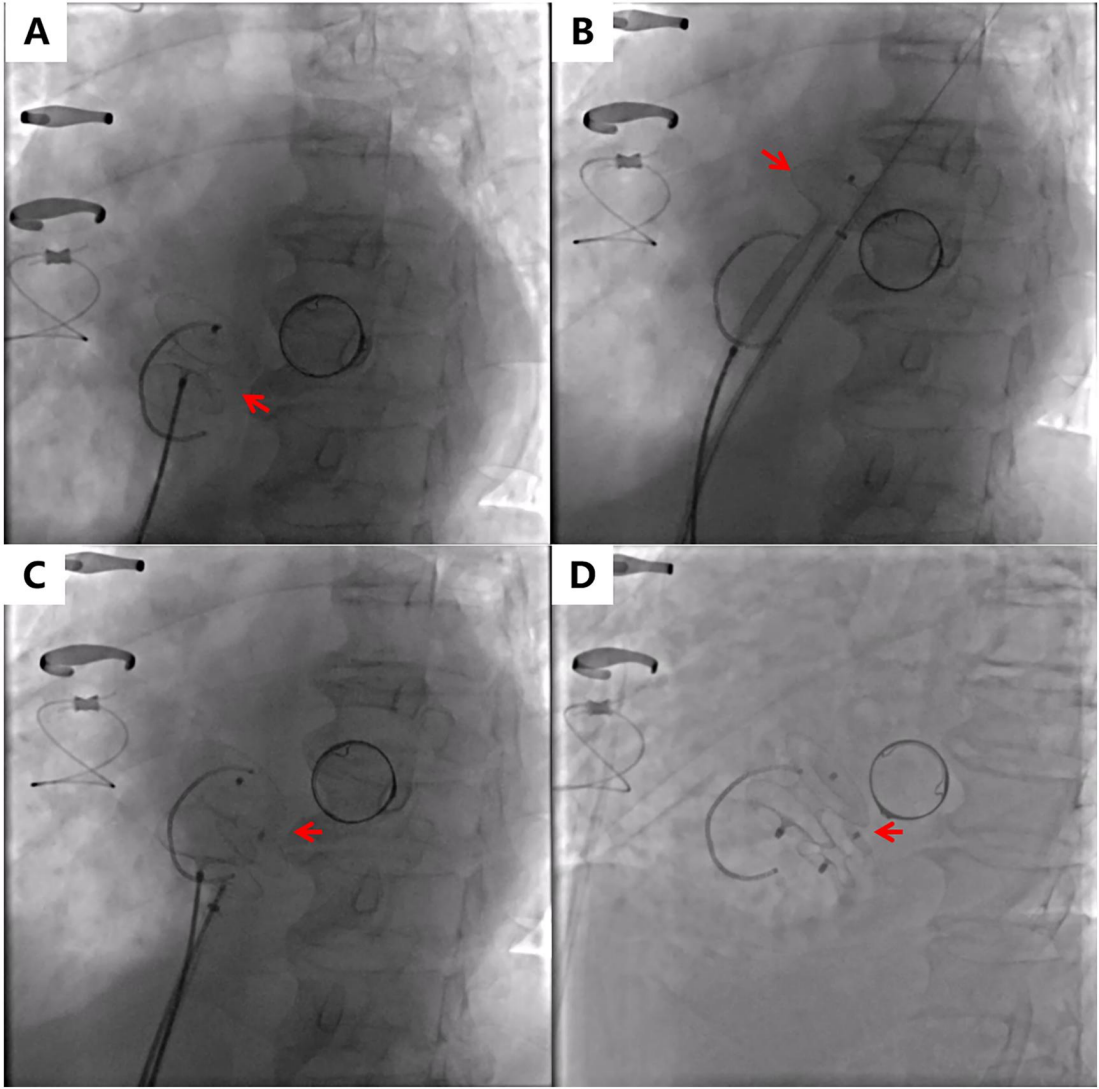
Fluoroscopic images demonstrating dual-device transcatheter closure of the iatrogenic atrial septal defect. **(A)** Deployment of the first 24-mm occluder; **(B)** Partial retrieval of the first device due to significant residual shunt and placement of the second delivery sheath; **(C)** Sequential release of the two occluders; **(D)** Final optimal positioning of both devices with no residual shunt.

The procedure was performed under fluoroscopic guidance combined with intraprocedural transthoracic echocardiography. Transesophageal echocardiography was not used because the patient had known esophageal varices. Intracardiac echocardiography was not available at our institution at the time of the procedure. Balloon sizing was not performed because the defect was an irregular, traction-related lesion following surgery, and balloon manipulation might have enlarged the tear or distorted the anatomy. Invasive Qp/Qs measurement was not performed because preoperative transthoracic echocardiography had already clearly demonstrated a large left-to-right shunt with direct evidence of right heart volume overload. Bilateral femoral venous access was obtained. A right heart catheter was advanced across the iASD into the left upper pulmonary vein under guidewire guidance. A super-stiff guidewire was positioned to secure the delivery pathway, after which a 12F delivery sheath was advanced into the left atrium. An initial attempt was made to deploy a 24-mm Asia-Pacific ASD occluder. Although the left atrial disc expanded satisfactorily, intraoperative echocardiography revealed a persistent large residual shunt surrounding the device. Therefore, a dual-device closure strategy was adopted. The first occluder was partially retrieved but left *in situ*. Using the same approach, a second guidewire and a 9F delivery sheath were advanced into the left atrium via the left femoral vein, and a 12-mm Asia-Pacific ASD occluder was positioned adjacent to the first device. Under digital subtraction angiography, both occluders were sequentially released. Final assessment confirmed stable positioning of both devices with complete elimination of residual shunting and no interference with the bioprosthetic mitral valve or adjacent structures (Figure 2).

**Figure 3:**
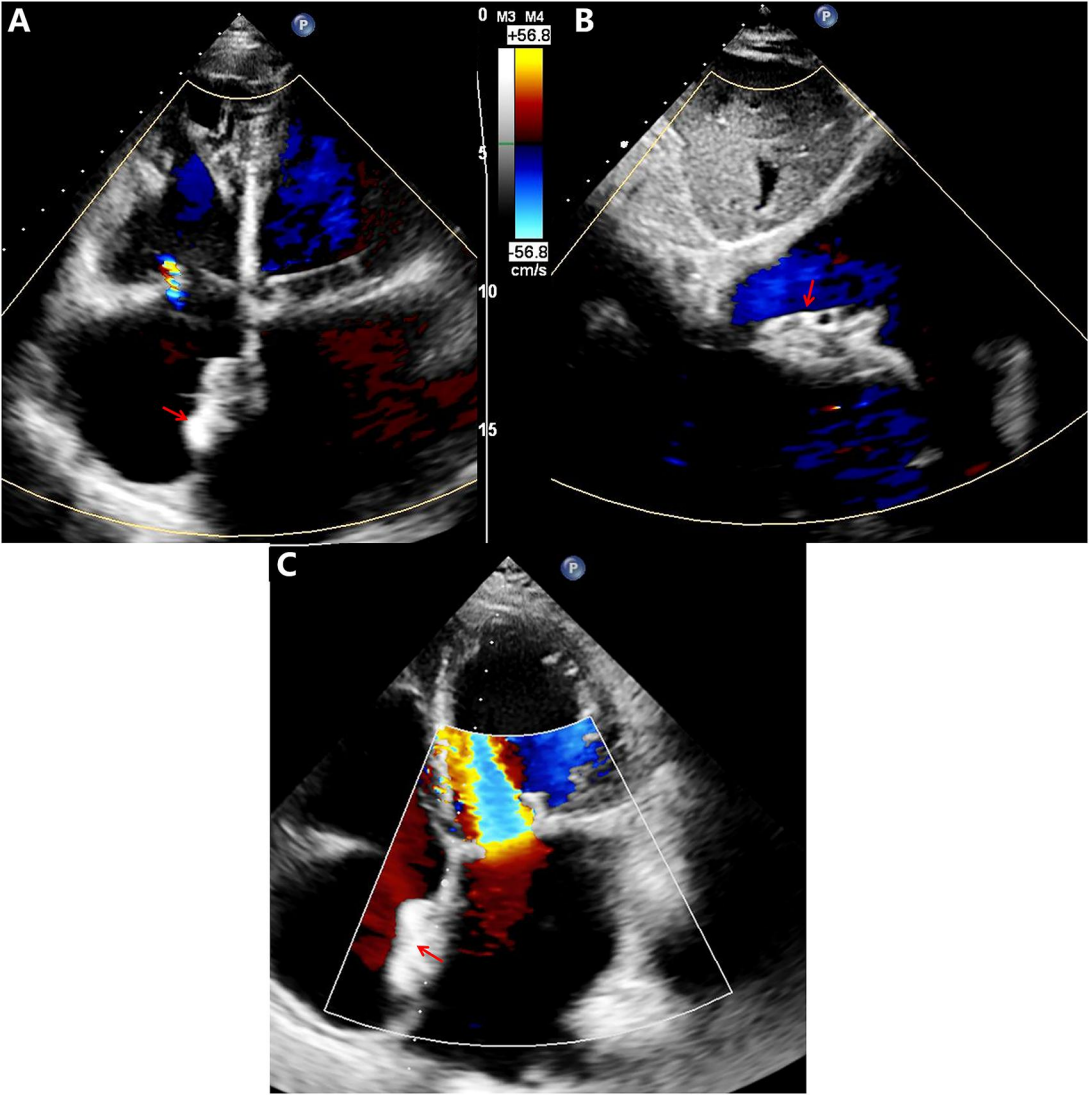
Postoperative and follow-up transthoracic echocardiography findings. **(A,B)** Color Doppler imaging in apical four-chamber and subcostal views demonstrates no residual interatrial shunt post-procedure; **(C)** Six-month follow-up confirms stable device position with complete elimination of interatrial flow.

The patient experienced no vascular access complications, arrhythmias, device embolization, or thromboembolic events following the procedure. A follow-up transthoracic echocardiogram (TTE) on postoperative day 3 confirmed stable positioning of both occluders, complete elimination of interatrial shunting, and marked reduction of tricuspid regurgitation (Figures 3A, B). The patient demonstrated significant clinical improvement, with dyspnea relieved to NYHA class I–II and notable reduction in peripheral edema. Considering his elevated bleeding risk, single-agent anticoagulation with rivaroxaban 15 mg daily was prescribed. The patient was discharged on postoperative day 4 with optimized heart failure and cirrhosis-related medical therapy. At 6-month follow-up, he remained clinically stable with resolution of heart failure symptoms, and echocardiography confirmed persistent device stability, complete closure of the interatrial shunt, and recovery of LVEF from 45% to within normal limits (Figure 3C).

## Discussion

Iatrogenic atrial septal defect (iASD) is a common sequela following structural cardiac surgery or catheter-based interventions requiring transseptal access. Although intentional atrial septotomy is typically closed at the end of surgery, postoperative re-opening may still occur due to suture loosening, disruption, or uneven septal tissue tension. Small defects are generally expected to close spontaneously, whereas larger defects rarely heal and may result in persistent left-to-right shunting, leading to right-sided volume overload, worsening tricuspid regurgitation, and increased pulmonary artery pressures (6, 7). Consequently, patients with a large persistent iASD may develop hemodynamic instability and congestive heart failure, often with a poor diuretic response (8). In addition, right-to-left shunting may result in hypoxemia, while bidirectional shunts have been shown to correlate with worse clinical outcomes (9). To date, high-quality prospective evidence and formal guidelines are lacking; thus, management remains individualized and guided by multidisciplinary heart team evaluation. Currently accepted indications for interventional closure include hypoxemia due to right-to-left shunting, documented or anticipated right-sided chamber volume overload and dysfunction, high risk of paradoxical embolism, and cryptogenic stroke (8, 9).

This case describes a large, persistent iASD that developed after bioprosthetic mitral valve replacement and was associated with progressive heart failure symptoms, fulfilling the criteria for interventional closure. Postoperative iASDs are typically located near the fossa ovalis, presenting with relatively regular morphology and adequate rims, which theoretically provide favorable anatomical conditions for transcatheter closure (10). However, when the defect results from a surgical atrial septotomy, the tear may be subject to greater tension, leading to a larger defect that requires selection of a correspondingly larger occluder. Oversized devices may interfere with prosthetic leaflet motion and valvular hemodynamics, thus increasing procedural complexity. Additionally, the patient had advanced liver disease with significant coagulopathy and thrombocytopenia, which markedly increased the risk of perioperative bleeding and hepatic decompensation, rendering reoperative surgical repair unsuitable. Based on multimodality imaging assessment and multidisciplinary heart team discussion, a dual-device percutaneous closure strategy was adopted. Previous studies have demonstrated that complex or multiple ASDs require individualized transcatheter planning based on defect size, spacing, septal rim adequacy, and atrial septal compliance, and may necessitate the use of multiple devices, customized device selection, or devices with larger discs and smaller waists (11). In one cohort of 50 such patients, procedural success was achieved in all cases, with mid- to long-term complete closure rates of approximately 92%, indicating that even complex atrial septal anatomy can be effectively and safely treated when optimal device selection and imaging-guided deployment strategies are applied. Although most reported cohorts focus on congenital ASDs, these findings remain clinically relevant for iatrogenic, incision-related defects.

In this case, a 24-mm occluder was initially selected in an attempt to achieve single-device closure; however, intraoperative echocardiography demonstrated a substantial residual shunt, suggesting that the iASD was likely a postoperative traction-related fissure or an irregular-rim defect, making complete sealing difficult with a conventional single-device approach. Under these circumstances, a dual-device strategy was adopted. By placing two devices in adjacent positions to achieve complementary coverage and rim reinforcement, this approach enhances closure completeness and long-term stability while avoiding the potential risk of prosthetic valve interference associated with selecting a significantly larger device. In this procedure, the first device was partially retrieved but left *in situ* to provide anchoring and positional support, followed by precise deployment of the second device. This ultimately resulted in complete closure without compromising prosthetic valve function. These findings indicate that a dual-device strategy may serve as a feasible, minimally invasive alternative for complex or large, irregular iASDs, particularly in patients who are unsuitable for surgical re-repair or who present with substantially elevated bleeding risk. This case has several limitations. Preprocedural computed tomography was not performed because the patient had concomitant renal impairment and was at risk of contrast-induced nephropathy. Therefore, defect morphology and rim assessment were based solely on transthoracic echocardiography. This may have underestimated the complexity of the iatrogenic atrial septal defect and, to some extent, contributed to the suboptimal result with the initial single-device closure, necessitating the dual-device strategy. In the future, when no contraindications exist, similar cases may benefit from three-dimensional computed tomography or three-dimensional transesophageal echocardiography for more precise preprocedural planning.

## Conclusion

This case highlights that percutaneous closure remains a safe and feasible therapeutic option for postoperative iASDs that are large, morphologically irregular, or have inadequate rim support, especially in patients with high surgical risk, coagulation disorders, or impaired baseline cardiac function. For complex iASDs in which complete sealing cannot be achieved with a single device, a dual-occluder strategy may serve as an effective alternative, providing improved anatomic conformity and device stability while avoiding structural interference associated with oversized devices. The successful outcome in this case provides a potentially valuable treatment reference for similar high-risk patient populations.

## Data Availability

The data underlying this article are available in the article

## Data availability statement

The original contributions presented in the study are included in the article/supplementary material, further inquiries can be directed to the corresponding authors.
